# The COVID-19 pandemic and food security in low- and middle-income countries: a review

**DOI:** 10.1186/s40066-022-00391-4

**Published:** 2022-12-02

**Authors:** Jeffrey R. Bloem, Jarrad Farris

**Affiliations:** 1 International Food Policy Research Institute, Washington, D.C., USA; 2 USA Department of Agriculture, Economic Research Service, Kansas City, MO USA

**Keywords:** COVID-19, Coronavirus pandemic, Food security, Income shocks, Markets, Trade, Q18, O2, I38, H5, F6

## Abstract

We review findings from the emerging microeconomic literature on observed changes in food insecurity associated with the COVID-19 pandemic. To do so, we focus our review on studies in low- and middle-income countries that include household survey data measuring food insecurity collected both before and after the onset of the COVID-19 pandemic. We first focus on several studies—seven from countries in Sub-Saharan Africa and one from India—that estimate immediate changes in food insecurity associated with the COVID-19 pandemic. Next, we review subsequent analysis studying longer term changes in food insecurity associated with the COVID-19 pandemic. This review, therefore, complements existing macroeconomic projections of food insecurity based on expected changes in income and prices.

## Introduction

The COVID-19 pandemic has led to widespread economic and social disruptions around the world. In addition to potential exposure to a contagious and deadly virus, job losses and reductions in earned income persist for a large share of the world’s population. Global poverty projections based on the World Bank’s PovcalNet and International Monetary Fund (IMF) data suggest that the number of people living below the $1.90 per day poverty line increased by at least 68 million, and the number of people living below the $3.20 per day poverty line increased by at least 140 million, in 2020 [53].^1^ Compared to pre-pandemic projections, expected GDP growth rates completely reversed, changing from an expected expansion to an expected contraction [10].

The USDA’s Economic Research Service (ERS) food security projections further highlight a large increase in the number of people experiencing food insecurity around the world due to the COVID-19 pandemic [9, 10]. The IFSA model projects per capita food demand—based on expected changes to income, prices, and food supply—and compares this projection with a nutritional target of 2100 cal per person per day, which according to the Food and Agriculture Organization (FAO) is a caloric level that is necessary to sustain a healthy and active lifestyle [23]. This projection provides estimated levels of food security and nutritional intake in 76 low- and middle-income countries around the world. In a follow-up article to the 2020 IFSA report, Baquedano et al. [10] update the 2020–2030 projections of global food security associated with the COVID-19 pandemic. These updated projections estimate that in 2020 the number of food-insecure people reached 921 million, an increase of 160 million from pre-pandemic projections. The 2021 IFSA report projects the prevalence of food insecurity in 2021 will increase by nearly an additional 294 million people [10].

The IFSA macroeconomic projections help illuminate the scale of the global consequences of the COVID-19 pandemic on food insecurity. They indicate a potential setback of the recent global progress toward meeting the United Nation’s Sustainable Development Goals and highlight a distinct challenge to ending hunger and achieving food security for all people by 2030 [34, 46]. These macroeconomic projections, however, are only designed to predict global, regional, and country-level changes in food insecurity, they are unable to provide insight on more nuanced, local-level, and within country changes in food insecurity associated with the COVID-19 pandemic.

In this review, we supplement these existing macroeconomic projections estimated with the IFSA model and presented in the IFSA report by discussing the emerging microeconomic literature that specifically tracks food insecurity among households measured both before and after the onset of the COVID-19 pandemic in low or middle-income countries. In doing so, we highlight local-level differences in food insecurity, that are not captured by the larger scale, macroeconomic projections. These insights include assessments of pandemic-related market disruptions, rural–urban differences, variation across socio-economic groups, and the effectiveness of social projection programs. The emerging microeconomic literature, however, is limited in geographic scope as detailed microeconomic data are only available in a small share of countries around the world. Taken together, insights from macroeconomic projections and the emerging microeconomic literature complement each other well and help inform public and private decision-makers about rapidly emerging changes in international food insecurity associated with the COVID-19 pandemic.

Our review leads to ten key takeaways, which include six lessons and four points of caution, and complements existing reviews by Santeramo and Dominguez [49] and Tabe-Ojong et al. [51]. We want to emphasize that our review here is complementary to these other existing reviews, and each of these reviews should be read and considered together. This paper continues as follows in  “Inclusion criteria” section, with a brief discussion about our inclusion criteria and methodology for our review. In  “Six early lessons” section we discuss the six lessons from the early studies that meet our inclusion criteria and study immediate changes in food insecurity associated with the COVID-19 pandemic. In “Points of caution” section, we discuss four points of caution with these early studies and comment on how these limitations influence the interpretation of the lessons from this emerging literature. In “Subsequent analysis of longer term changes in food insecurity” section, we review more recent studies that analyze longer term changes in food insecurity associated with the COVID-19 pandemic. Finally, “Concluding thoughts” section concludes.

## Inclusion criteria

The COVID-19 pandemic influenced many aspects of life—including health, education, consumer behavior, work, etc.—for people all around the world. Food security represents an important outcome that could, conceptually, be influenced by the COVID-19 pandemic in several ways. On the demand side, the COVID-19 pandemic led many people to lose their jobs and a meaningful share of their household’s income. This could influence the ability of vulnerable households to purchase enough food. On the supply side, the COVID-19 pandemic disrupted supply chains and, in some places at some times, led to a reduction in a sufficient supply of food. In addition, some countries closed schools for an extended period during the first year of the COVID-19 pandemic, which removed a means, whereby many young children around the world receive food each week. Each of these mechanisms, and many others, demonstrate possible ways in which the COVID-19 pandemic could have influenced food security. Our aim in writing this review is to summarize the lessons from the literature so as to better understand these possible mechanisms based on analysis of data documenting real-life experiences during the COVID-19 pandemic.

Our review includes two inclusion criteria. First, our review is restricted to studies in low- and middle-income countries. This is done for two reasons: (i) to supplement the existing projections of the IFSA model which includes 76 low- and middle-income countries and (ii) because, while much has been written about food insecurity during the COVID-19 pandemic in the United States and other high-income countries [4, 30, 49, 55], relatively little is known about changes in food insecurity in low- and middle-income countries despite widespread concern [7, 25, 38, 47].

Second, we focus on studies that analyze survey data measuring food insecurity from both before and after the onset of the pandemic. We focus first on several studies—seven from countries in Sub-Saharan Africa and one from India—that estimate immediate changes in food insecurity associated with the COVID-19 pandemic [1–3, 5, 16, 33, 37, 41]. We next review several more recent studies [18, 42, 43, 48] that study longer term changes in food insecurity associated with the COVID-19 pandemic, to assess if and how well the preliminary lessons hold when considering a longer study time period. Other relevant studies that fall outside of these inclusion criteria are also discussed and help contextualize and explain the findings in this emerging literature. This is done to provide as detailed an understanding of the immediate and short-term changes in food insecurity amid the COVID-19 pandemic as possible at the time of writing this review.

Given that the literature documenting changes in food insecurity associated with the COVID-19 pandemic is relatively new, our review of the literature required a careful approach of monitoring working paper series and recently published journal articles. The studies included in our review are either recently published—in the *American Journal of Agricultural Economics, Food Policy*, and *World Development*—or posted as lightly peer reviewed working papers in the National Bureau of Economic Research (NBER) working paper series, the International Food Policy Research Institute (IFPRI) working paper series, or the USDA’s ERS COVID-19 Working Paper Series.

## Six early lessons

In this section, the six cross-cutting lessons from the emerging microeconomic literature on changes in food insecurity associated with the COVID-19 pandemic are discussed. Specific parts of Table 1 are referred to throughout our review of these studies, which documents key information and the headline finding of each of the studies that meet our inclusion criteria. Table 1 summarizes each of the reviewed articles by reporting information about the geographic area and time frame of the study, the data source, the outcome variable measuring food insecurity, the empirical method used, the key finding of the study, and four questions assessing specific mechanisms underlying the results.

**Table 1:** Summary of studies on the COVID-19 pandemic and food insecurity

	Abay et al. [1]	Adjognon et al. [2]	Aggarwall et al. [3]	Amare et al. [5]	Ceballos et al. [16]	Kansiime et al. [37]	Mahmud and Riley [41]	Hirvonen et al. [33]
A: Published?	IFPRI Discussion Paper	Food Policy	NBER Working Paper	IFPRI Discussion Paper	World Development	World Development	World Development	American Journal of Agricultural Economics
B: Geographic area	Rural Ethiopia	Mali	Rural Liberia and rural Malawi	Nigeria	Haryana and Odisha, India	Kenya and Uganda	Rural Uganda	Addis Ababa, Ethiopia
C: Geographically representative?	No	Yes	No	Yes	No	No	No	Yes
D: Data source	Phone survey from ongoing project	LSMS and follow-up phone survey^i^	Phone survey from ongoing project	LSMS and follow-up phone survey	Phone survey from ongoing project	Online survey	Phone survey from ongoing project	Phone survey from ongoing project
E: Pre-survey date	March–August 2019	October 2018–July 2019	January 2020	July 2018–February 2019	April 2020	Pre-pandemic recall	March 2020	August–September 2019
F: Post-survey date	June 2020	May–June 2020	August 2020	April–May 2020	May 2020	April 2020	May 2020	May–August 2020
G: Short-term results?	Yes	Yes	Yes	Yes	Yes	Yes	Yes	Yes
H: Empirical method	Difference-in-differences^ii^	Pre–post comparison and difference-in-differences	Panel data with fixed effects	Difference-in-differences	Pre–post comparison	Pre–post comparison	Pre–post comparison	Pre–post comparison and difference-in-differences
I: Outcome variable	Food gap^iii^	Food Insecurity Experience Scale (FIES)^iv^	Diet diversity, hunger scale, and food consumption	Partial Food Insecurity Experience Scale (FIES)	Food availability and access indicators	Food Insecurity Experience Scale (FIES)	Food expenditures per adult equivalent	Food consumption and diet diversity
J: Key finding	*Increase* in food insecurity	*Increase* in food insecurity	*No change* in food insecurity	*Increase* in food insecurity	*Mixed* results	*Increase* in food insecurity	*Increase* in food expenditures	*No change* in food insecurity
K: Do pandemic-related disruptions explain the result?^v^	Yes	Yes	N/A, markets disrupted but food insecurity remained stable	Yes	Yes	Yes	Yes	N/A, income and job loss but food consumption remained stable
L: Do results differ in urban vs. rural areas	N/A	Yes	N/A	No	N/A	N/A	N/A	N/A
M: Do results differ by socio-economic status?	N/A	N/A	N/A	Yes, more adverse changes for poorer households	N/A	N/A	Yes, more adverse changes for wealthier households	N/A
N: Do results differ by access to social support?	Yes, Productive Safety Net Program (PSNP)	N/A	Yes, cash transfers	N/A	N/A	N/A	N/A	N/A

This list includes the authors’ tabulation of studies that analyze an outcome variable measuring some dimension of food insecurity over time with measures pre-dating the pandemic and measures collected after the onset of the pandemic. Many studies, which we discuss in this article, do not meet these criteria

^i^The Living Standards Measurement Study (LSMS) is a series of household surveys conducted by the World Bank

^ii^A difference-in-difference regression specification is like a pre–post comparison, but the pre–post difference is combined with a difference across two groups

^iii^The “food gap” is the number of months the household was not able to satisfy its food needs [13]

^iv^The Food Insecurity Experience Scale (FIES) is a measurement tool used to estimate the extent of the multidimensional experience of food insecurity [50]

^v^Pandemic-related disruptions can include government-mandated lockdowns or individual behavior change due to fear of contracting COVID-19

### Food insecurity increases amid the COVID-19 pandemic

Row J in Table 1 reports the key finding for each of the studies that meet our inclusion criteria. Five studies find evidence of increasing food insecurity associated with the COVID-19 pandemic [1, 2, 5, 37, 41]. Two studies find no evidence of changes in food insecurity associated with the COVID-19 pandemic [3, 33]. The existence or absence of food security is a multidimensional concept. Commonly, food security is considered to have been achieved when each of four interrelated components are met: availability (i.e., physical supply of food at a local or national level), access (i.e., affordability of food in sufficient quantity), utilization (i.e., meeting of all nutritional needs), and stability (i.e., uninterrupted ability to meet food needs) [52]. In the following discussion, we highlight the core findings of these studies are and make note of the specific dimension(s) of food security measured by each study.

First, studying rural households in the highland regions of Ethiopia, Abay et al. [1] use phone survey data from an ongoing project and find that, compared to survey responses in March–August 2019, the fraction of households reporting that they are not able to satisfy their food needs increased by June 2020. In addition, the authors find that these households report an increase in the number of months in which they are not able to satisfy their food needs amid the COVID-19 pandemic. As this measure of food security lets the household define what their food needs are, this change in food insecurity cannot be attributed to a specific food security dimension. Abay et al. [1] also show that this adverse change in food insecurity is virtually offset by participation in Ethiopia’s Productive Safety Net Program. This is discussed in more detail in "The role of social protection programs" section.

Second, using nationally representative data from Mali, Adjognon et al. [2] find that moderate food insecurity—as measured using the Food Insecurity Experience Scale (FIES)—increased between a pre-pandemic household survey and a phone survey implemented 3 months after the first recorded cases of COVID-19 in Mali.^2^ The FIES is specifically designed to measure the food access dimension of food security [8]. As discussed in “Differences between rural and urban areas” section, when reviewing differences in observed changes between rural and urban areas, Adjognon et al. [2] find that this measured change in food insecurity is almost entirely driven by changes within urban areas, with very little change observed within rural areas. In addition, Adjognon et al. [2] observe that these contrasting changes in food insecurity between urban and rural areas are plausibly explained by deeper and more dramatic initial pandemic-related disruptions in Mali’s urban areas compared to rural areas.

Third, in a related study, Amare et al. [5] use nationally representative data from Nigeria and compare changes in food insecurity, measured with an abbreviated FIES scale, over time between geographic areas with high vs. low pandemic-related disruptions.^3^ Amare et al. [5] find that households in areas with relatively high levels of pandemic-related disruptions are more likely to experience food insecurity. Amare et al. [5] implement the most direct analysis investigating the role of pandemic-related disruptions in influencing observed changes in food insecurity associated with the COVID-19 pandemic. The authors find that Nigerian states with higher recorded COVID-19 case counts and with stricter lockdowns experienced larger adverse changes in food insecurity associated with the pandemic than other Nigerian States.

Fourth, using non-representative data from an online survey in Kenya and Uganda, Kansiime et al. [37] estimate that food insecurity—specifically the food access dimension as measured using the FIES—worsened in the first 2 months of the COVID-19 pandemic compared to recall data from prior to the pandemic. Kansiime et al. [37] presents a more limited set of results than the other studies that meet our inclusion criteria due to their use of non-representative data from an online survey and recall data to record pre-pandemic information.

Finally, using data collected in May 2020, Mahmud and Riley [41] follow-up with rural households in Uganda who were interviewed in person in March 2020 to examine short-term changes in livelihood indicators associated with the pandemic. Mahmud and Riley [41] find evidence of a substantial decline in non-farm income which households respond to by reducing their food expenditures. This expenditure-based measure of food security relates to the access dimension of food security. Mahmud and Riley [41] present some of the clearest evidence that the largest changes in food insecurity associated with the COVID-19 pandemic may not be concentrated among the poorest households.

Two studies find no evidence of changes in food insecurity associated with the COVID-19 pandemic, despite finding evidence of dramatic disruptions to incomes and agricultural markets [3, 33]. Both studies use a combination of food security measures which cover the access and utilization dimensions of food security. First, following up on rural households that were participants in a cash transfer experiment in both Liberia and Malawi, Aggarwal et al. [3] do not find any evidence of changes in food insecurity—as measured with a household dietary diversity score, a household hunger scale, and household food consumption—associated with the COVID-19 pandemic. Despite observing no measurable adverse change in food insecurity associated with the COVID-19 pandemic on average, Aggarwall et al. [3] find that the receipt of cash transfers—an increasingly popular social protection program in low- and middle-income countries—improves the food security of rural households in both Liberia and Malawi. Second, using panel data of urban households in Addis Ababa, Ethiopia, Hirvonen et al. [33] also do not find any evidence of changes in food insecurity—as measured with a household dietary diversity scale and household food consumption—associated with the COVID-19 pandemic. In contrast to other countries in the region, Ethiopia did not enforce as strict of a pandemic-motivated lockdown. Ethiopia’s relatively stable food security measure provides some evidence that relatively greater lockdown restrictions have a negative impact on food insecurity.

In addition, one study finds mixed results across the two Indian states of Haryana and Odisha [16]. Studying households in the two Indian states of Haryana and Odisha, Ceballos et al. [16] find that households in Haryana experienced large and adverse changes in food insecurity—measured by asking respondents if food was sufficiently available and affordable—while households in Odisha experienced no measurable increase in food insecurity associated with the COVID-19 pandemic. These findings, which focus on the availability and access dimensions of food security, highlight why microeconomic analysis can help supplement macroeconomic projections. As the results found by Ceballos et al. [16] make clear, changes in food insecurity associated with the coronavirus pandemic may differ dramatically within countries. These studies highlight the food security resiliency, at least in the relatively short term, of some households amidst major pandemic-related economic disruptions.

### Pandemic-related disruptions in food markets and earned income

Row K in Table 1 reports whether each study that meets our inclusion criteria finds evidence that the changes in food insecurity are associated with pandemic-related disruptions in markets and earned income. In some countries, national or local governments implemented policies with the objective of slowing the spread of the COVID-19 virus. These policies could be a factor that explain observed differences across countries. Josephson et al. [36] use the nationally representative Living Standard Measurement Study (LSMS) data collected by the World Bank to calculate statistics documenting public knowledge of COVID-19 virus containment policies and personal behaviors that can reduce the risk of contracting the virus. Public knowledge of both national COVID-19 virus containment policies as well as healthy personal behaviors are relatively high in Ethiopia, Nigeria, and Uganda but relatively low in Malawi [36]. Rows J and K in Table 1 show that all of the studies that find evidence of increased food insecurity and meet our inclusion criteria, also find evidence of pandemic-related disruptions that plausibly explain the increased measure of food insecurity associated with the COVID-19 pandemic.

Of all the studies summarized in Table 1, Amare et al. [5] performs the most in-depth analysis on how pandemic-related disruptions influence changes in food insecurity associated with the COVID-19 pandemic. The authors estimate changes over time and between states with high levels of recorded COVID-19 cases vs. low levels of recorded COVID-19 cases. In an alternative set of analyses, the authors also estimate changes over time between states with high levels of lockdown measures vs. states with low levels of lockdown measures, which they validate with Google mobility data.^4^ In both sets of analyses, Amare et al. [5] find that changes in food insecurity are more dramatic in states with more COVID-19 cases and with higher levels of lockdown measures.

In a similar study, Adjognon et al. [2] find that pandemic-related disruptions—as measured by recorded COVID-19 case and death counts, Google mobility data, and self-reported behavior—were much more dramatic in Mali’s urban areas compared to Mali’s rural areas. Consistent with the idea that the measured changes in food insecurity are associated with the intensity of pandemic-related disruptions, Adjognon et al. [2] find that households in urban areas experienced larger changes in food insecurity on average than households in rural areas of Mali. The other studies that find evidence of increasing food insecurity associated with the COVID-19 pandemic also find evidence that pandemic-related disruptions may plausibly explain these changes [1, 16, 37, 41].

The mixed results found by Ceballos et al. [16] can also be plausibly explained by the presence of pandemic-related disruptions in food supply chains and markets. Ceballos et al. [16] find that households in Haryana, India experienced an increase in food insecurity while households in Odisha, India did not experience an increase in food insecurity. This difference in food insecurity changes before and during the COVID-19 pandemic coincides with a larger observed shock to food supply in Haryana than in Odisha.

Disruptions to the supply of food, and associated price effects, represent one reason why strictly enforced lockdown measures may influence food insecurity. India's national lockdown, beginning on March 24, 2020 and extending for 21 days, represents one of the most strictly enforced national lockdowns in the world. Despite a declining pre-pandemic price trend, Narayanan and Saha [44] examine price data of 22 commodities from over 100 market centers in India and document rising prices since the country's lockdown. The authors also survey 50 food retailers who report operational challenges associated with sourcing inventory. In addition, Lowe et al. [40] find that food arrivals in India’s food wholesale markets fell dramatically and food wholesale prices increased in 3 weeks following India’s national lockdown. Six weeks after India’s lockdown, however, food arrivals and prices had fully recovered and reverted to pre-pandemic levels. The evidence documented by Narayanan and Saha [44] and Lowe et al. [40] highlight how a strict lockdown, like the one implemented by India, can lead to deep short-term changes in food supply and food prices. However, even in the case of India’s strict lockdown, Lowe et al. [40] shows that the food supply chain was relatively resilient after an initial disruption.

### Some evidence of resiliency

Even in studies that do not find any change in food insecurity associated with the COVID-19 pandemic [3, 33], there is evidence of substantial pandemic-related disruptions (Table 1, Rows J and K). These points imply that some sub-populations have been relatively resilient, at least in terms of food security, to the adverse shocks to earned income and prices associated with the COVID-19 pandemic.

In particular, although Aggarwall et al. [3] do not find any evidence of changes in food insecurity associated with the COVID-19 pandemic among rural households from Liberia and Malawi, the authors find evidence that the pandemic severely disrupted market activity, resulting in relatively large declines in income among market vendors. Similarly, although Hirvonen et al. [33] do not find any evidence of changes in food insecurity associated with the COVID-19 pandemic among urban households in Addis Ababa, Ethiopia, the authors do find evidence of dramatic reductions in income and job losses associated with the pandemic. In contrast to many other East African countries, Ethiopia never implemented a strict lockdown. Therefore, despite reductions in income and job losses, the food supply chain in Addis Ababa remained resilient throughout the first few months of the COVID-19 pandemic. Taken together, these results highlight a caveat to existing macroeconomic projections estimating an increase in the number of food insecure people based on expected changes to income and prices. The relationship between earned income and food security is not the same for all people within a given country. Among some sub-populations in some countries, despite dramatic reductions in earned income associated with the COVID-19 pandemic, food security has remained resilient. There are several factors that influence the relationship between income and food security which are not easily incorporated into macroeconomic projections and this highlights the complementary nature of supplementing existing macroeconomic projections with microeconomic analysis.

### Differences between rural and urban areas

There is conflicting evidence on potential food insecurity differences between urban and rural areas (Table 1, Row L). On one hand, Adjognon et al. [2] find that changes in food insecurity associated with the COVID-19 pandemic are much larger in Mali’s urban areas than in Mali’s rural areas. On the other hand, Amare et al. [5] do not find any difference in food insecurity associated with the COVID-19 pandemic between Nigeria’s urban and rural areas.

The potential difference in changes in food insecurity associated with the COVID-19 pandemic between urban and rural areas may be related to differences in how urban and rural households experience market disruptions. For instance, Narayanan and Saha [44], Lowe et al. [40], and Wiseman [54] document changes in food supply and increased food prices associated with market disruptions from the COVID-19 pandemic. These changes may have differing implications for food insecurity depending on whether households are net-buyers or net-sellers of food. For instance, although analysis by Josephson et al. [36] of data from Ethiopia, Malawi, Nigeria, and Uganda shows only weak evidence of more reductions of income in urban areas than in rural areas, net-buyers of food bear the burden of higher food prices and rural households may be able to grow the food they consume, highlighting the potential for more dramatic changes in food insecurity associated with the COVID-19 pandemic in urban areas compared to rural areas.

Focusing on Mali, Adjognon et al. [2] document three observations suggesting that disruptions driven by the pandemic may have been more intense in urban areas—particularly Mali's capital city of Bamako—compared to rural areas. First, recorded COVID-19 case and death counts are dramatically skewed toward Bamako. Although these statistics almost certainly underestimate the true incidence of COVID-19 infections and deaths in Mali, they are indicators that influence containment policy efforts and motivate concern among individuals of contracting the virus within Bamako. Second, Google mobility data show that individuals in Bamako have adjusted their time spent in every geographic location category more than individuals in Mali as a whole.^5^ Finally, urban respondents to phone surveys are more likely to report making pandemic-related health choices—such as washing hands more than usual, avoiding gatherings with physical contact, and avoiding gatherings with more than ten people—than rural respondents. Taken together, these details may partially explain why Mali’s urban areas may have had larger changes in food insecurity associated with the COVID-19 pandemic than Mali’s rural areas. Mali is a country with already high levels of food insecurity, particularly in rural areas. Therefore, at least in the relative short-term, the COVID-19 pandemic may have reduced the rural–urban food insecurity gap by being disproportionately more disruptive in urban areas relative to rural areas.

Additional evidence of differential changes in food insecurity associated with the COVID-19 pandemic between urban and rural areas comes from contrasting the results of Hirvonen et al. [33] and Abay et al. [1] who both study households in Ethiopia. Although Hirvonen et al. [33] find no change in food consumption and diet diversity among urban households in Addis Ababa, Ethiopia, Abay et al. [1] find a decrease in the food gap, an indicator of food shortfall at the household level, among rural households in Ethiopia. On the surface, comparing these two results suggests that changes in food insecurity associated with the COVID-19 pandemic may be more dramatic in Ethiopia's rural areas compared to Ethiopia's capital city of Addis Ababa. This conclusion contrasts with the findings of Adjognon et al. [2] from Mali and could be driven by several factors. First, highlighting potential differences across geographic areas, the food supply chain in Addis Ababa, Ethiopia may be more resilient than the food supply chain in Bamako, Mali. Second, the population studied by Abay et al. [1], covering particularly drought-prone rural regions of Ethiopia, may represent a particularly vulnerable population that is more prone to large, adverse changes in food insecurity. Finally, these differences could be driven by variation in the outcome variables measuring food insecurity in each study: food consumption and diet diversity by Hirvonen et al. [33], food gap by Abay et al. [1], and FIES by Adjognon et al. [2].

Narratives about differential changes in food insecurity associated with the COVID-19 pandemic must confront existing nuance about the role of geographic location-specific features that influence food insecurity. Differential changes in food insecurity between urban and rural areas associated with the COVID-19 pandemic remains difficult to predict across countries. For example, Aggarwal et al. [3] find no change in diet diversity—a household hunger scale—and food consumption among households in the rural areas of Liberia and Malawi. Similarly, Hirvonen et al. [33] find no change in food consumption and diet diversity among urban households in Addis Ababa, Ethiopia, while Abay et al. [1] find an increase in food insecurity—measured by the food gap—among a selected sample of rural households in Ethiopia. In addition, Mahmud and Riley [41] find evidence of a decrease in food expenditures among rural households in Uganda. The mixed evidence on changes in food insecurity between urban and rural areas associated with the COVID-19 pandemic may also relate to the changing dynamics of the spread of the COVID-19 virus. For example, in the United States, the consequences of the pandemic seemed to first materialize in major metropolitan areas, perhaps due to population density and propensity for travel. Over time, the effects tended to spread into rural areas, which by some measures, ended up being even more deeply disrupted [20].

### Differences by socio-economic status

Two included studies find evidence of differential changes in food insecurity associated with the COVID-19 pandemic by socio-economic status [5, 41] (Table 1, Row M). These studies, however, do not lead to a clear narrative about how changes in food insecurity associated with the pandemic may vary across socio-economic groups.

As the COVID-19 virus began to spread around the world, many researchers and analysts predicted that the consequences of the COVID-19 pandemic may depend critically on household characteristics, such as existing vulnerabilities to income shocks and food insecurity [6, 12, 19]. Conceptually, however, it is not clear how different levels of socio-economic status may differentially influence changes in food insecurity associated with the COVID-19 pandemic. On one hand, it may seem plausible that poorer households are more vulnerable, due to limited access to financial safety nets and being less able to guard themselves from the disruptions driven by the pandemic. On the other hand, wealthier households may be more integrated into the national or global economic system and may be more directly affected by pandemic-related disruptions.

Three cases highlight that, at least in the relative short-term, there is mixed evidence on whether the poorest households experience the largest adverse changes in food insecurity associated with the COVID-19 pandemic [3, 5, 41]. First, Aggarwal et al. [3] find no evidence of worsening food insecurity associated with the pandemic in either rural Liberia or rural Malawi. In fact, the authors find a modest decrease in food insecurity measures in rural Malawi, which is likely due to the fortunate timing of the harvest season coinciding with the COVID-19 pandemic. Therefore, the households observed by Aggarwal et al. [3] seem to be more insulated from any market disruptions due to the availability of locally produced food and did not experience an increase in food insecurity as a result. By contrast, market vendors observe relatively large declines in their income in the first few months of the COVID-19 pandemic [3]. Second, analysis by Mahmud and Riley [41] finds that households that are more reliant on non-farm income, such as enterprise or salaried income, experienced larger declines in income. This finding emphasizes that the changes in food insecurity associated with the COVID-19 pandemic are not necessarily largest for the poorest households. In the context of rural Uganda, Mahmud and Riley [41] note that the relatively wealthy households experienced the largest increases in food insecurity associated with the COVID-19 pandemic. Finally, and to the contrary, Amare et al. [5] show that pandemic-related shutdown policies implemented in Nigeria are associated with larger changes in food insecurity among those who live in more remote regions, in areas with relatively high levels of conflict, and poorer households.

A clear assessment of how the effect of the COVID-19 pandemic differs across individuals and households in different socio-economic groups is lacking from the emerging literature. Future research to fill this gap would do well to disentangle competing factors relating to the mediating role of poverty between the COVID-19 pandemic and food insecurity. On one hand, households living in poverty will typically be more vulnerable—due to a less robust financial safety net—to experiencing food insecurity in the aftermath of the negative shocks to income and employment driven by the COVID-19 pandemic. On the other hand, as shown by Bargain and Aminjonov [11], individuals living in poverty in low- and middle-income countries may be less likely to reduce their mobility for work-related activities, may be less connected economically to negative global income shocks, and thus may be less likely to experience income declines in the first place.

### The role of social protection programs

Two studies specifically estimate the role of a specific social protection program in mitigating any adverse change in food insecurity associated with the COVID-19 pandemic [1, 3]. Both studies find evidence suggesting that these social protection programs—Ethiopia’s Productive Safety Net Program [1]) and cash transfers in rural Liberia and Malawi [3]—help mitigate the observed adverse change in food insecurity among these sub-populations (Table 1, Row N).

Studying rural households in Ethiopia, Abay et al. [1] provide evidence supporting the protective role of social safety net programs amidst the COVID-19 pandemic. They show that participation in Ethiopia's Productive Safety Net Program, a rural food security program based on cash and in-kind food payments, offsets most of the adverse change in food insecurity associated with the pandemic. Similarly, studying rural households in Liberia and Malawi, Aggarwall et al. [3] find that households who received cash transfers experienced improved food security—measured with a dietary diversity scale and with a food consumption score—amid the pandemic. Cash transfer programs, however, are not a panacea. Gentilini et al. [27] provide a global review of social protection measures implemented thus far and note that, although informal sector workers tend to be a main target of cash transfer programs implemented in response to the COVID-19 pandemic, not all of these workers successfully received this financial assistance. Furthermore, while countries' pandemic-related cash transfer programs tended to be large relative to pre-pandemic levels, they also tended to be of relatively short duration. Providing effective social and economic support for households that experience the deepest and most dramatic consequences of the COVID-19 pandemic will need to overcome a host of design, targeting, and implementation challenges [28].

In the face of adverse economic shocks, and in the absence of effective policy responses, households typically seek to limit adverse consequences via a suite of coping strategies, including reliance on savings or borrowing, informal sector work, selling of assets, and migration. The 2008 financial crisis highlights some of the ways that households and individuals use existing formal (e.g., credit and insurance from financial institutions) and informal mechanisms (e.g., social insurance from family, friends, and community-based organizations) to cope with adverse shocks [32]. Although the pre-existence of these coping mechanisms may allow for resiliency among some sub-populations, the adverse health and economic shocks associated with the COVID-19 pandemic are far reaching. Unlike the 2008 financial crisis and other similar widespread macroeconomic shocks, pandemic-related income reductions may not allow for some of these common coping strategies. For instance, government policies to curtail the spread of COVID-19 through mobility restrictions (e.g., lockdowns) as well as personal best practices to reduce exposure risk (e.g., social distancing) may make informal sector work and migration infeasible [28]. This could be particularly consequential in low- and middle-income country contexts, where the informal sector is a major source of employment or migration to urban settings to seek informal employment is common particularly in response to adverse economic shocks [26, 31, 39]. In Kenya and Uganda, for example, over three quarters of urban and rural employment is in the informal sector [37].

In the case of Uganda, which implemented strict lockdown measures, Mahmud and Riley [41] find that rural households tend to respond to the adverse income shock of the pandemic in three ways. First, households reduce food consumption. Mahmud and Riley [41] find that food expenditures per adult equivalent fell by around 40% and the percentage of households that reported missing at least one meal a month rose from 30% to 52%. Second, households use up available savings and borrow more, but avoid liquidating fixed assets and selling livestock. Third, households increase total household labor supply to own farm crop and livestock activities. Taken together, Mahmud and Riley [41] argue their findings suggest that these households are reducing consumption and relying on savings and borrowing to prevent irreversible economic consequences from the COVID-19 pandemic. Selling off productive assets could more fully alleviate food insecurity concerns in the short term, but at the expense of future asset accumulation and a weakened ability to respond to future shocks. A greater reliance on own farm activities further suggests an increase in subsistence-based agriculture as well as a reduction in off-farm opportunities. These households face a dilemma. Reducing short-term food consumption creates health consequences that worsen the longer the strategy persists. Nevertheless, selling limited assets to allow for greater food consumption in the short-term may leave households even more vulnerable in the long-term.

## Points of caution

Having so far reviewed six lessons from the emerging microeconomic literature on changes in food insecurity associated with the COVID-19 pandemic, we now discuss four points of caution which help frame how to interpret and extrapolate the insights discussed above. These points of caution identify gaps in this emerging literature. Table 1 includes information about the geographic scope of each study, the study time frame, the empirical methods used, and the key outcome variable measuring food insecurity.

### Limited geographic scope

The geographical scope of the data used by each of the studies that meet our inclusion criteria is shared in Row C of Table 1. Given the limited availability of detailed microeconomic panel data collected amid a global pandemic, the geographic scope of the emerging microeconomic literature is extremely limited.^6^ Only one study that meets our inclusion criteria examines a geographic area outside of Sub-Saharan Africa [16]. At the same time, of the remaining studies—all of which focus on a specific country within Sub-Saharan African—only two, Adjognon et al. [2] in Mali and Amare et al. [5] in Nigeria use a nationally representative data source. The rest focus on sub-populations in specific sub-regions of countries, such as rural areas of Liberia and Malawi [3], rural Uganda [41], rural Ethiopia [1], Addis Ababa, Ethiopia [33], or report findings using non-representative data [37].

The limited geographic scope of available microeconomic data that collect panel data on measures of food insecurity both before and after the onset of the COVID-19 pandemic limits our knowledge of how food insecurity changed in association with the pandemic. This limited geographic scope is problematic, because important differences in food insecurity are observed across countries. Although 97.7% of the population in Eritrea are estimated to be food insecure, based on the macroeconomic projections from the IFSA model [10], the more nuanced, local-level patterns of changes in food insecurity associated with the COVID-19 pandemic in this country are not known. Similarly, although updates to the macroeconomic income and price data did not change the projected level of food insecurity in both the Democratic Republic of Congo and Senegal, based on the macroeconomic projections from the IFSA model [10], the specific reasons that food security remains resilient—at least on average—within these countries is not known.

### Only short-term evidence

The emerging literature is only able to investigate immediate and short-term changes in food insecurity associated with the COVID-19 pandemic. Beyond long-term macroeconomic projections, very little is known about any changes in food insecurity in the longer term. In fact, it is likely that the changes in food insecurity discussed from the studies reviewed in this article will not persist in the medium and long term.

Along with changes in the spread and intensity of the pandemic, policy responses and households' coping strategies will also evolve overtime. For example, Adjognon et al. [2] find that the increases in food insecurity are larger in urban areas compared to rural areas in Mali. This likely represents the more dramatic short-term disruption of the pandemic in Mali's urban areas compared to rural areas. As has already been observed in the United States, as the pandemic progresses some pandemic-related disruptions may become more dramatic in rural areas compared to urban areas [20]. This suggests that short-term effects are not necessarily indicative of the medium- or long-term effects.

Based on the evidence presented in the early studies reviewed so far, very little is known about the specific pattern that pandemic-related consequences will take in the medium or long term. For example, some evidence using antibody COVID-19 tests suggests that in countries such as Kenya, Malawi, and Mozambique large shares of the population have already been exposed to the COVID-19 virus [45]. However, data limitations in these studies themselves limit the ability to conclude that the worst of any pandemic-related consequences are in the past anywhere in the world. As the short-term changes in food insecurity associated with the pandemic transcend into the medium and even long-term, future research will need to similarly shift to longer term outcomes.

### Methodological challenges

The empirical method used by each of the studies examined here are reported in Row H of Table 1. These methods range from more simple pre–post comparisons using panel data to more sophisticated difference-in-differences regression specifications.^7^ Due to the nature of the COVID-19 pandemic, which to some extent influenced the entire world in some way, credible causal identification of the impact of the pandemic on food insecurity—among many other outcomes—is particularly challenging. There is no obvious comparison in the data to a group that has not experienced some form of disruption from the COVID-19 pandemic. This is a limitation of all studies in this emerging literature, and other adjacent literatures studying the economic consequences of the COVID-19 pandemic, to date [29]. Without reliable data on COVID-19 infection rates, it is difficult to understand the overall extent of the spread of the virus and which geographical areas and communities have been most deeply affected by the pandemic. This limits the ability to disentangle the effect of the pandemic from, for example, the effects of seasonality or within-country agro-ecological variation, such as rainfall or temperature, or conflict. Despite these limitations, analysis of changes in food insecurity associated with the COVID-19 pandemic provide useful insights that can be used by policymakers in the short, medium, and long-term aftermath of the pandemic around the world.

### Different measures of food insecurity across studies

The primary outcome variable, or variables, used to measure food insecurity in each of the studies that meet our inclusion criteria is reported in Row I of Table 1. Three studies use the Food Insecurity Experience Scale (FIES) which asks a series of questions that aim to elicit a household's lived experience with food insecurity [2, 5, 37]. The other studies use a variety of indicators that proxy for food insecurity, such as dietary diversity and food consumption [3, 33], food expenditures [41], the food gap [1], and food access [16]. This variety of survey tools used to measure food insecurity make clear comparisons between studies challenging.

Food security is a complex concept that often looks different in different geographical areas around the world. The FAO uses a broad definition of food security that highlights the multi-dimensional nature of the concept. According to the FAO, food security exists when, “all people, at all times, have physical, social, and economic access to sufficient, safe, and nutritious food that meet their dietary needs and food preferences for an active and healthy lifestyle” [22, 24]. Although this definition of food security is widely accepted, challenges persist in consistently measuring food security across time and space [15]. Despite this challenge, it remains possible to learn lessons from this emerging literature if care is taken to not make unfounded comparisons of the specific magnitude of changes in food insecurity associated with the pandemic across studies. The direction of these changes, and if changes are measurable at all, are more reasonably comparable across the studies than the magnitudes of such changes.

## Subsequent analysis of longer term changes in food insecurity

We now turn to a brief review of subsequent analysis that aim to build on the set of studies we review above. In particular, this analysis aims to address our second point of caution—that existing research is limited to immediate or short-term changes in food insecurity—by studying longer term changes in food insecurity associated with the COVID-19 pandemic.

There are several studies that meet our first inclusion criterion of studying food insecurity in low- and middle-income countries, but do not meet our second criterion of analyzing survey data measuring food insecurity from both before and after the onset of the pandemic. For example, Mueller et al. [43] study Bangladesh, Kenya, and Nigeria but only use data from October 2020 through April 2021. Similarly, Dasgupta and Robinson [18] study Armenia, Cambodia, Chad, Djibouti, Ethiopia, Malawi, Mali, Nigeria, South Africa, and Uganda over a variety of time periods, but do not include pre-pandemic data in their analysis. Finally, Maredia et al. [42] study Kenya, Zambia, Mali, Nigeria, and Senegal but only use data from September 2020 through November 2020. By relying on survey data collected after the onset of the COVID-19 pandemic, these studies are only able to provide snapshots of longer term changes in the food security status of households in these countries during the COVID-19 pandemic. As documented by the studies reviewed above, and as we show in Table 1, food insecurity increased dramatically in the initial months of the pandemic. Therefore, to assess changes in food insecurity associated with the COVID-19 pandemic, one must be able to make comparisons to pre-pandemic levels of food insecurity.

Thus, we focus our discussion of longer term food insecurity changes associated with the COVID-19 pandemic on the work of Rudin-Rush et al. [48], because, at the time of writing this review, it is the only longer term study that meets both of our inclusion criteria. Using data collected by the World Bank’s Living Standards Measurement Study–Integrated Surveys on Agriculture (LSMS–ISA) Initiative, Rudin-Rush et al. [48] analyze changes in food insecurity in Burkina Faso, Ethiopia, Malawi, and Nigeria from prior to the COVID-19 pandemic up to one full year after its onset. While this study is limited in geographic scope, as it is driven by the availability of household level panel data measuring food insecurity both before and after the onset of the COVID-19 pandemic, it allows for a useful assessment of if and how well the preliminary lessons discussed above may hold over a longer period.

Rudin-Rush et al. [48] document three main findings about longer term changes in food insecurity associated with the COVID-19 pandemic. First, in each of the four countries and consistent with evidence from other countries, there is an initial spike in food insecurity in the early months of the COVID-19 pandemic. This initial spike is followed by a gradual decline, but as of the end of 2021, levels of food insecurity have not returned to levels observed prior to this initial spike.

Second, Rudin-Rush et al. [48] find that, for most measures in all four countries, food insecurity increased more in rural areas than in urban areas during the first year of the COVID-19 pandemic. This finding, perhaps, helps settle some ambiguity discussed above from the studies analyzing immediate changes in food insecurity associated with the COVID-19 pandemic. In particular, and as discussed above, Adjognon et al. [2] find that food insecurity declined more in Mali’s urban areas relative to rural areas using data from the first 3 months of the COVID-19 pandemic. To the contrary, Amare et al. [5] do not find any difference between changes in food insecurity associated with the COVID-19 pandemic between rural and urban areas in Nigeria. The longer term changes in food insecurity between rural and urban areas documented by Rudin-Rush et al. [48] seem to follow the changing dynamics of the spread of COVID-19 and associated socio-economic disruptions over time. In particular, although the virus and pandemic-related disruptions first fell most heavily on urban areas, over time the virus and associated disruptions spread to rural areas, where the consequences were more severe.

Finally, to approximate household vulnerability and socio-economic status, Rudin-Rush et al. [48] examine differences in changes in food insecurity between female-headed and male-headed households. The authors do not find evidence of differences in food insecurity changes between these two types of households in Burkina Faso, Ethiopia, or Malawi. In Nigeria, the authors find limited evidence that male-headed households experienced larger adverse changes in food insecurity relative to female-headed households. As discussed above, the studies analyzing immediate changes in food insecurity associated with the COVID-19 pandemic do not find consistent results when investigating changes between households at different levels of socio-economic status. The Rudin-Rush et al. [48] analysis of longer term changes in food insecurity associated with the COVID-19 pandemic provides little additional clarity on this point. This may be due to the competing dynamics associated with household socio-economic status. For example, less wealthy households may be more vulnerable to increased food insecurity due to being less able to weather the adverse socio-economic consequences of the pandemic while also being partially insulated from adverse food insecurity effects as a result of being less connected to and dependent on market and supply chain fluctuations for their day-to-day livelihoods.

## Concluding thoughts

In this article, we review the emerging microeconomic literature on changes in food insecurity associated with the COVID-19 pandemic in low- and middle-income countries. Our review is focused on studies that help supplement the macroeconomic projections discussed in the IFSA report using microeconomic survey data collected in a low- or middle-income country during the pandemic with at least one wave of survey data collected prior to the onset of the pandemic.

Our review leads to ten key takeaways, including six lessons and four points of caution, each of which are summarized in Table 1. First, the six lessons include the following: (i) most, but not all, studies find evidence of increasing food insecurity amid the COVID-19 pandemic (row J in Table 1). (ii) Increased food insecurity appears to be associated with pandemic-related disruptions in food markets and earned income (row K in Table 1). (iii) Despite evidence of pandemic-related disruptions across all studies (row K in Table 1), there is evidence of resilience, at least in terms of food security, among some sub-populations (row J in Table 1). (iv) Studies that meet our inclusion criteria and compare changes in food insecurity over time between rural and urban areas find conflicting results (row L in Table 1). (v) Studies that meet our inclusion criteria and compare changes in food insecurity over time between socio-economic groups find conflicting results (row M in Table 1). (vi) Studies that meet our inclusion criteria and examine the role of social protection programs find that these programs help mitigate any observed adverse change in food insecurity associated with the COVID-19 pandemic (row N in Table 1).

Second, the four points of caution include the following: (i) existing microeconomic data are limited in geographic scope. Two studies use nationally representative data and one study uses data representative of a large urban area. The remaining studies use data from specific sub-populations within a specific geographical area (row C in Table 1). (ii) All of the studies that meet our inclusion criteria examine immediate or short-term changes in food insecurity associated with the COVID-19 pandemic (rows E, F, and G in Table 1). (iii) Most studies use cutting-edge empirical methods that remain limited as the widespread consequences of the COVID-19 pandemic make finding a valid comparison group within the available data difficult (row H in Table 1). (iv) The outcome variable measuring food insecurity differ across many studies, which complicates direct comparisons across studies (row I in Table 1).

We also review the limited existing research on longer term changes in food insecurity associated with the COVID-19 pandemic which finds that the initial spike in food insecurity at the onset of the pandemic was followed by a gradual decline, but as of the end of 2021, levels of food insecurity have not returned to levels observed prior to this initial spike. It is not the intention of this article to provide a final analysis on the relationship between the COVID-19 pandemic and food insecurity. As we discuss throughout this review, although this emerging literature makes several contributions, there are many remaining questions left to be considered. Filling the gaps in the existing literature will require a considerable amount of effort and commitment from researchers across academic disciplines but doing so is necessary to understand the potential consequences of the COVID-19 pandemic that contribute to food insecurity and hunger.

## Footnotes

1 More information about the World Bank’s PovcalNet is available here: http://iresearch.worldbank.org/PovcalNet/introduction.aspx

2 The Food Insecurity Experience Scale (FIES) is a survey tool developed by the FAO to measure food insecurity based on the direct experiences of people relating to food security [8, 14, 50]. This experience-based measure of food insecurity offers greater precision than other measures of food insecurity that rely on country-level food supply estimates [17, 50].

3 Specifically, Amare et al. [5] use the following three indicators of food insecurity from the FIES: “Household members had to skip a meal because there was not enough money or other resources to get food?” “Household members ran out of food because there was not enough money or other resources to get food?” and “Household members have not eaten all day because of a lack of money or other resources?” As in other studies that use FIES, these questions focus on the food access dimension of food security.

4 Additional details about Google's Community Mobility Reports are available at https://www.google.come/covid19/mobility/.

5 These geographic location categories include: Grocery and pharmacy, retail and recreation, parks, transportation. stations, workplaces, and residential.

6 A limited geographic scope is not unique to the emerging microeconomic literature on changes in food insecurity associated with the COVID-19 pandemic. Indeed, existing macroeconomic projections rely on data that fills missing data by interpolating and predicting data based on the available data and historic trends [35]. Additionally, over half of the papers presented at the 2017 Northeast Universities Development Conference use microeconomic data from just four countries [21], an observation that persists at other leading academic conferences and in academic journals that publish development economics research.

7 A difference-in-difference regression specification is like a pre-post comparison, but the pre-post difference is combined with a difference across two groups.
